# The phenotypic and molecular spectrum of PEHO syndrome and PEHO-like disorders

**DOI:** 10.1093/brain/awx155

**Published:** 2017-07-12

**Authors:** Vincenzo Salpietro, Massimo Zollo, Jana Vandrovcova, Mina Ryten, Juan A Botia, Veronica Ferrucci, Andreea Manole, Stephanie Efthymiou, Fuad Al Mutairi, Enrico Bertini, Marco Tartaglia, SYNAPS Study Group, Henry Houlden

**Affiliations:** 1 Department of Molecular Neuroscience, Institute of Neurology, UCL Institute of Neurology, London WC1N 3BG, UK; 2 Department of Molecular Medicine and Medical Biotechnologies “DMMBM”, University of Naples “Federico II”, Naples 80131, Italy; 3 CEINGE Biotecnologie Avanzate, Naples 80131, Italy; 4 European School of Molecular Medicine, SEMM, University of Milan, Italy; 5 King Saud bin Abdulaziz University for Health Sciences, Department of Pediatrics, Division of Genetics, Riyadh 14611, Saudi Arabia; 6 Genetics and Rare Diseases Research Division, Ospedale Pediatrico “Bambino Gesù”, Rome 00146, Italy

Sir,

We read with great interest the article recently published in *Brain* by Anttonen *et al.* (2017) reporting a homozygous p.Ser31Leu mutation in the *ZNHIT3* gene causing PEHO (progressive encephalopathy with peripheral oedema, hypsarrhythmia, and optic atrophy) syndrome (MIM 260565) in a series of affected children from Finland.

PEHO was first described in the Finnish population (Salonen *et al.*, 1991) and the diagnostic criteria (Supplementary Table 1) were initially formulated by Somer in 1993 (Somer, 1993; Somer *et al.*, 1993). While the description of PEHO in siblings born from healthy and sometimes consanguineous parents supports autosomal recessive inheritance (Riikonen, 2001), from a genetic point of view PEHO syndrome has remained a poorly understood condition until recently.

The affected Finnish children reported by Anttonen *et al.* showed the classical clinical and radiological features of PEHO syndrome and were all found to harbour a founder p.Ser31Leu homozygous mutation in *ZNHIT3*, suggesting a common ancestral origin of these families. Supporting this Finnish founder effect, in the ExAC database containing 60 706 individuals (last accessed April 2017) the p.Ser31Leu variant represents the most frequent single nucleotide polymorphism in *ZNHIT3* (rs148890852), being carried by 30 (of a total of 39) subjects from a cohort of 3298 Finnish individuals [minor allele frequency (MAF): 0.0045; carriers frequency: 0.009]. Notably, in all remaining ethnic groups the variant is present at a much lower frequency with only five carriers from 31 694 (non-Finnish) Europeans (MAF: 7.888 × 10^−5^; carrier frequency: 0.0001), and it is (almost) absent in some other populations (e.g. Asian, Latino).

The *ZNHIT3* gene is likely to be important in the PEHO group of disorders from different populations, we therefore analysed our whole-exome and whole-genome data from a cohort of well-defined PEHO syndrome (*n = *9) and ‘PEHO-like’ syndrome (*n = *15) cases from variable (non-Finnish) ancestries and failed to identify additional mutations in *ZNHIT3*. Although a rare condition, based on the carrier frequency data from publicly available databases and on the screening analysis from our disease cohort, we suggest that autosomal recessive PEHO syndrome due to *ZNHIT3* mutations is likely to be exceptionally rare outside of Finland (especially in association with the isolated founder p.Ser31Leu mutation).

There are many examples of genetic isolated in families from Finland (Sajantila *et al.* 1996; Pakkasjärvi *et al.*, 2006). This is because the Finnish population have an isolated ancestry in a small founder group, followed by several historical bottle neck events that have led to genetic drift and enrichment of certain rare and low frequency variants that are almost absent in other ethnicities (Abecasis *et al.*, 2012; Lemmelä *et al.*, 2016). Although PEHO could be considered a very rare syndrome, it has been reported worldwide, including from Australia (Field *et al.*, 2003), Turkey (Tekgül and Tütüncüoğlu, 2000), Japan (Fujimoto *et al.*, 1995), South America (Caraballo *et al.*, 2011) and different European countries (Nieto-Barrera *et al.*, 2003; Klein *et al.*, 2004); overall, non-Finnish patients account for approximately half of the cases published to date (Langlois *et al.*, 2016; Anttonen *et al.*, 2017).

These data suggest genetic heterogeneity, a well-known scenario in many neurodevelopmental and neurodegenerative disorders caused by mutations in different genes implicated in overlapping conditions characterized by severe progressive encephalopathy and epilepsy (Noebels *et al.*, 2012). In addition, certain broad phenotypes associated with the PEHO main features should be also considered based on previous clinical reports of patients lacking any of the mandatory diagnostic criteria that have been diagnosed with ‘PEHO-like’ disorders (Chitty *et al.*, 1996; Field *et al.*, 2003; Pavlidou *et al.*, 2016). In this regard, an autosomal dominant *de novo* mutation in *CDKL5* has been previously identified in a single proband with a PEHO-like disorder (Gawlinski *et al.*, 2016) and a recent report described a young female with PEHO syndrome harbouring a *de novo* missense mutations in *KIF1A* (Langlois *et al.*, 2016). Furthermore, a homozygous truncating mutation of the *CCDC88A* gene was recently found as a cause of PEHO-like syndrome in a consanguineous family and the authors identified a similar neurological phenotype in the *Ccdc88a* knock-out mouse presenting progressive microcephaly and corpus callosum deficiency (Nahorski *et al.*, 2016).

Importantly, we recently characterized a new autosomal recessive neurodevelopmental and degenerative disorder caused by biallelic mutations in *PRUNE1*, with some of the affected individuals showing PEHO or PEHO-like features (Zollo *et al.*, 2017). *PRUNE1* is a member of the DHH (Asp-His-His) phosphoesterase and exopolyphosphatase protein superfamily of molecules important for cell motility, and implicated in cancer progression (Tammenkoski *et al.*, 2008). Of interest, we found that pathogenic mutations cause enhancement of PRUNE1 enzymatic exopolyphosphatase (PPase/PPX) activity and mutated PRUNE1 colocalizes with microtubules during mitosis to form mitotic spindle via binding to α/β-tubulin and also demonstrated that PRUNE1 mutants cause a delay in microtubule polymerization, particularly affecting the nucleation-phase (Zollo *et al.*, 2017; Ferrucci *et al.*, unpublished results).

In one of the reported families from our original study (Family C), we identified a homozygous p.D106N mutation involving an aspartic acid residue of the active and conserved DHH motif (Fig. 1A–C). Interestingly, the phenotype of the affected siblings from this family fulfilled the diagnostic criteria for PEHO syndrome, including severe hypotonia with onset shortly after birth and profound global developmental delay, early loss of visual fixation (Fig. 1D and E) and optic atrophy, normal head circumference at birth evolving to progressive microcephaly (Fig. 1F and G), infantile epileptic encephalopathy with hypsarrhythmia, and progressive CNS atrophy mainly involving the cerebellum and the brainstem (Fig. 1H and I). In addition, probands from the family showed some of the typical distinctive facial features (e.g. narrow forehead, short nose and open mouth appearance; Fig. 1D–G), and variably presented many of the additional supportive criteria for the diagnosis of PEHO, including a progressive white matter involvement on brain MRI (Fig. 1J and K), facial and/or limb oedema, abnormal brainstem auditory evoked potentials, brisk tendon reflexes in early childhood and slowing of the peripheral nerve conduction velocity in late infancy.

**Figure 1:**
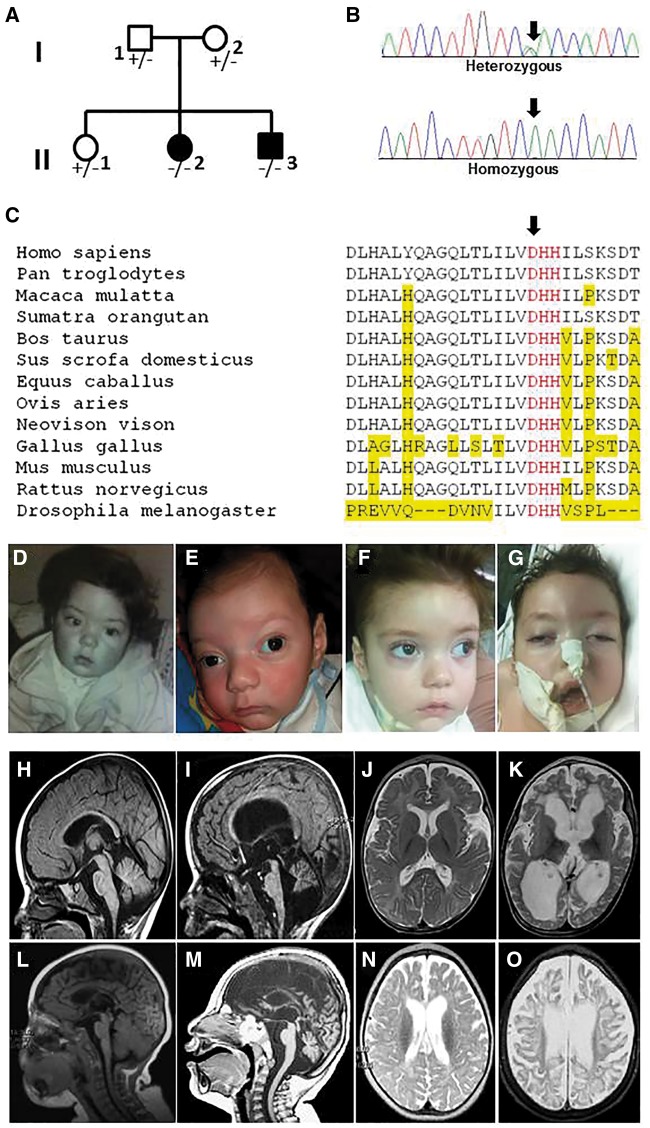
**Family tree, Sanger sequencing, multiple-sequence alignment of *PRUNE* p.D106N family, and clinico-radiological natural history of *PRUNE1-* and *TBCD-* associated tubulinopathies with PEHO and PEHO-like features.** (**A**) Pedigree from the Family carrying the p.D106N change in PRUNE1 (corresponding to Family C from Zollo *et al.*, 2017). (**B**) Electropherograms of carrier parent and affected sibs from the family. (**C**) Multiple-sequence alignment showing complete conservation of DHH motif (in red) of PRUNE1 orthologues across species. The aspartic acid residue replaced by the p.D106N mutation is indicated with an arrow; non-conserved residues are underlined in yellow. (**D**) Patient II-2 at 4 months of age, note the early loss of visual fixation.(**E**) Patient II-3 at 2 months of age, note the early loss of visual fixation. (**F** and **G**) Patient II-3 at the age of 16 months; note the hypotonia and distinctive facial features, including narrow forehead and open mouth appearance. Brain MRIs showing sagittal views of Patient II-3 at 6 months (**H**) and 16 months of age (**I**) show progressive global brain atrophy but more specifically there is evidence of cerebellar and brain stem atrophy. Axial views images in the same patient (II-3) at 6 months (**J**) and 16 months of age (**K**) shows progressive diffuse white matter abnormalities along with progressive brain atrophy. Brain MRIs performed in two affected siblings carrying the homozygous p.P1122L change in TBCD showing sagittal views from the first patient (Patient F118_347; from Flex *et al.*, 2016) at the age of 8 months (**L**) and his affected brother (Patient F118_347) at the age of 2 years (**M**). There is progression of CNS atrophy from early mild cortical atrophy and thin corpus callosum to (**L**) to cortical and subcortical brain atrophy mainly involving cerebellum (vermis and folia) and the brainstem (**M**). Axial views of the two siblings also show progression of white matter involvement (**N** and **O**).

As the most important neuropathology features in PEHO syndrome are classically observed at the cerebellar level due to severe loss of granule cells and abnormal Purkinje cells, we analysed the PRUNE1 protein expression in a developing mouse cerebellum in order to better understand the role of *PRUNE1* in the disease. In this regard, immunofluorescence analyses show that murine PRUNE1 protein is strongly expressed along the entire cerebellum collected at postnatal Day P1 (Fig. 2A), either in the granular layers or in the developing Purkinje cells, with particular enrichment of the expression in Purkinje cells migrating to the cerebellar surface (Fig. 2B). Taken together, present and previous data suggest the importance of PRUNE1 during the proliferation, migration and differentiation processes of granular and Purkinje neuron precursor cells, possibly also through an effect on microtubules dynamic during cell division (Carotenuto *et al.*, 2006; Zollo *et al.*, 2017).

**Figure 2:**
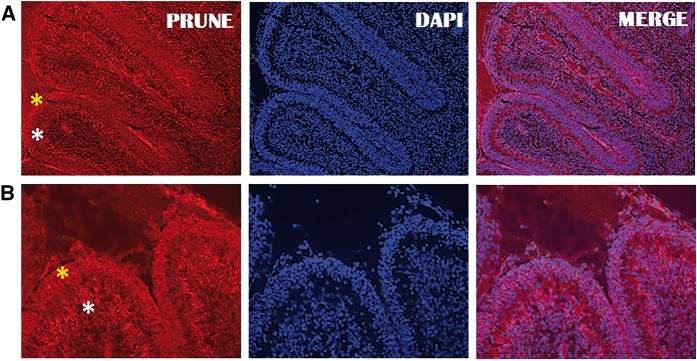
**Prune-M1 expression during cerebellum development.** Sagittal sections of cerebellum of mice collected at postnatal Day P1 were stained with an antibody against endogenous murine PRUNE1 (Prune-M1). Yellow asterisks show cerebellar granular layers and white asterisks show Purkinje cells. The antibody was detected with a tetramethylrhodamine (TRICT)-conjugated secondary antibody (as shown in red, see **A***left* and **B***left*). DAPI (4', 6-diamidino-2-phenylindole) was used for DNA staining (as shown in blue, **A***middle* and **B***middle*). The overlay between Prune-M1 and DAPI is shown in **A** and **B***right*. The higher magnification photomicrographs in **B** show Prune-M1 is expressed in all of the layers (in both Purkinje and granular layers) of the early developed cerebellum. Scale bars = 50 μm (**A**) and 100 μm (**B**).

Interestingly, the *CCDC88A* gene recently associated to PEHO-like syndrome with polymicrogyria (MIM 260565) encodes girdin, a protein involved in postnatal neural development and cancer progression that has also been suggested to play an important role in cytoskeleton organization, via either a possible direct association to microtubules (Simpson *et al.*, 2005) or a localization (and interaction) with a number of cytoskeleton- and microtubule-binding proteins (Ota *et al.*, 2013). Furthermore, another PEHO-like phenotype variably characterized by hypotonia, severe psychomotor delay, infantile epileptic encephalopathy with optic atrophy, abnormal visual and brainstem evoked potentials, brisk tendon reflexes, microcephaly, CNS progressive atrophy (mainly affecting cerebellum and brainstem) with white matter involvement (Fig. 1L–O), has been recently described in a number of families in association with biallelic mutations of *TBCD,* a gene encoding one of the five co-chaperones required for assembly of the α/β-tubulin heterodimer that is crucial in cytoskeleton organization (Flex *et al.*, 2016; Miyake *et al.*, 2016).

It is uncertain if mutations in the *ZNHIT3* share the disease mechanisms associated to tubule dysfunction; using knockdown and genome editing experiments in zebrafish embryos and knockdown experiments in mouse cerebellar neurons, Anttonen *et al.* showed that *ZNHIT3* is essential in cerebellar granule neuron survival and migration. Of interest, results from a large interactome network analysis recently identified a direct interaction between ZNHIT3 and the cytoskeleton associated protein 5, encoded by *CKAP5* (Hein *et al.*, 2015); however, further studies will be needed to assess a possible role of *ZNHIT3* in cytoskeleton assembly and organization.

In conclusion, the recent characterization of new autosomal recessive disorders associated with defects in proteins that regulate cytoskeleton microtubule dynamics and influence neuronal migration imply heterogeneity in the PEHO spectrum of disorders and highlight a Finnish founder effect in the *ZNHIT3* gene. Thus, we suggest that in addition to *ZNHIT3* the screening of additional disease-causing genes (including *PRUNE1*, *TBCD* and *CCDC88A*) should be considered in patients presenting with PEHO or PEHO-like features.

## Supplementary Material

Supplementary Table 1

## References

[awx155-B1] Abecasis GR, Auton A, Brooks LD, DePristo MA, Durbin RM, Handsaker RE, An integrated map of genetic variation from 1,092 human genomes. Nature 2012; 491: 56–65. 23128226 10.1038/nature11632 PMC3498066

[awx155-B2] Anttonen AK, Laari A, Kousi M, Yang YJ, Jaaskelainen T, Somer M, , ZNHIT3 is defective in PEHO syndrome, a severe encephalopathy with cerebellar granule neuron loss. Brain 2017; 140: 1267–79. 28335020 10.1093/brain/awx040

[awx155-B3] Caraballo RH, Pozo AN, Gomez M, Semprino M PEHO syndrome: a study of five Argentinian patients. Pediatr Neurol 2011; 44: 259–64. 21397166 10.1016/j.pediatrneurol.2010.11.007

[awx155-B4] Carotenuto P, Marino N, Bello AM, D'Angelo A, Di Porzio U, Lombardi D, PRUNE and NM23-M1 expression in embryonic and adult mouse brain. J Bioenerg Biomembr 2006; 38: 233–46. 17033939 10.1007/s10863-006-9044-z

[awx155-B5] Chitty LS, Robb S, Berry C, Silver D, Baraitser M PEHO or PEHO-like syndrome? Clin Dysmorph 1996; 5: 143–52. 8723564 10.1097/00019605-199604000-00006

[awx155-B6] Field MJ, Grattan-Smith P, Piper SM, Thompson EM, Haan EA, Edwards M, PEHO and PEHO-like syndromes: report of five Australian cases. Am J Med Genet A 2003; 122A: 6–12. 12949965 10.1002/ajmg.a.20216

[awx155-B7] Flex E, Niceta M, Cecchetti S, Thiffault I, Au MG, Capuano A, Biallelic mutations in TBCD, encoding the tubulin folding cofactor D, perturb microtubule dynamics and cause early-onset encephalopathy. Am J Hum Genet 2016; 99: 962–73. 27666370 10.1016/j.ajhg.2016.08.003 PMC5065658

[awx155-B8] Fujimoto S, Yokochi F, Nakano M, Wada Y Progressive encephalopathy with edema, hypsarrhythmia, and optic atrophy (PEHO syndrome) in two Japanese siblings. Neuropediatrics 1995; 26: 270–2. 8552220 10.1055/s-2007-979771

[awx155-B9] Gawlinski P, Posmyk R, Gambin T, Sielicka D, Chorazy M, Nowakowska B, PEHO syndrome may represent phenotypic expansion at the severe end of the early-onset encephalopathies. Pediatr Neurol 2016; 60: 83–7. 27343026 10.1016/j.pediatrneurol.2016.03.011 PMC5125779

[awx155-B10] Hein MY, Hubner NC, Poser I, Cox J, Nagaraj N, Toyoda Y, A human interactome in three quantitative dimensions organized by stoichiometries and abundances. Cell 2015; 163: 712–23. 26496610 10.1016/j.cell.2015.09.053

[awx155-B12] Klein A, Schmitt B, Boltshauser E Progressive encephalopathy with edema, hypsarrhythmia and optic atrophy (PEHO) syndrome in a Swiss child. Eur J Paediatr Neurol 2004; 8: 317–21. 15542387 10.1016/j.ejpn.2004.08.006

[awx155-B13] Langlois S, Tarailo-Graovac M, Sayson B, Drögemöller B, Swenerton A, Ross CJ, *De novo* dominant variants affecting the motor domain of KIF1A are a cause of PEHO syndrome. Eur J Hum Genet 2016; 24: 949–53. 26486474 10.1038/ejhg.2015.217 PMC4867456

[awx155-B14] Lemmelä S, Solovieva S, Shiri R, Benner C, Heliövaara M, Kettunen J, Genome-wide meta-analysis of sciatica in Finnish population. PLoS One 2016; 11: e0163877. 27764105 10.1371/journal.pone.0163877 PMC5072673

[awx155-B15] Miyake N, Fukai R, Ohba C, Chihara T, Miura M, Shimizu H, Biallelic TBCD mutations cause early-onset neurodegenerative encephalopathy. Am J Hum Genet 2016; 99: 950–61. 27666374 10.1016/j.ajhg.2016.08.005 PMC5065661

[awx155-B16] Nahorski MS, Asai M, Wakeling E, Parker A, Asai N, Canham N, CCDC88A mutations cause PEHO-like syndrome in humans and mouse. Brain 2016; 139(Pt 4): 1036–44. 26917597 10.1093/brain/aww014 PMC4806221

[awx155-B17] Nieto-Barrera M, Nieto-Jiménez M, Díaz-Fernandez F, Campaña-Marchal C, Candau Fernández-Mensaque R Progressive encephalopathy with oedema, hypsarrhythmia and optic atrophy (PEHO syndrome). A case report. Rev Neurol 2003; 36: 1044–6. 12808501

[awx155-B18] Noebels JL, Avoli M, Rogawski MA, Olsen RW, Delgado-Escueta AV Jasper's basic mechanisms of the epilepsies, 4th edn. Bethesda, MD: National Center for Biotechnology Information (US); 2012. 22787592

[awx155-B19] Ota H, Hikita T, Nishioka T, Matsumoto M, Ito J, Asai N, Proteomic analysis of Girdin-interacting proteins in migrating new neurons in the postnatal mouse brain. Biochem Biophys Res Commun 2013; 442: 16–21. 24211587 10.1016/j.bbrc.2013.10.126

[awx155-B20] Pakkasjärvi N, Ritvanen A, Herva R, Peltonen L, Kestilä M, Ignatius J Lethal congenital contracture syndrome (LCCS) and other lethal arthrogryposes in Finland—an epidemiological study. Am J Med Genet A 2006; 140A: 1834–9. 16892327 10.1002/ajmg.a.31381

[awx155-B21] Pavlidou E, Salpietro V, Phadke R, Hargreaves IP, Batten L, McElreavy K, Pontocerebellar hypoplasia type 2D and optic nerve atrophy further expand the spectrum associated with selenoprotein biosynthesis deficiency. Eur J Paediatr Neurol 2016; 20: 483–8. 26805434 10.1016/j.ejpn.2015.12.016

[awx155-B22] Riikonen R The PEHO syndrome. Brain Dev 2001; 23: 765–9. 11701291 10.1016/s0387-7604(01)00283-2

[awx155-B23] Sajantila A, Salem AH, Savolainen P, Bauer K, Gierig C, Pääbo S Paternal and maternal DNA lineages reveal a bottleneck in the founding of the Finnish population. Proc Natl Acad Sci USA 1996; 93: 12035–9. 8876258 10.1073/pnas.93.21.12035 PMC38178

[awx155-B24] Salonen R, Somer M, Haltia M, Lorentz M, Norio R Progressive encephalopathy with edema, hypsarrhythmia, and optic atrophy (PEHO syndrome). Clin Genet 1991; 39: 287–93. 2070547 10.1111/j.1399-0004.1991.tb03027.x

[awx155-B25] Simpson F, Martin S, Evans TM, Kerr M, James DE, Parton RG, A novel hook-related protein family and the characterization of hook-related protein 1. Traffic 2005; 6: 442–58. 15882442 10.1111/j.1600-0854.2005.00289.x

[awx155-B26] Somer M Diagnostic criteria and genetics of the PEHO syndrome. J Med Genet 1993: 30: 932–6. 8301648 10.1136/jmg.30.11.932 PMC1016602

[awx155-B27] Somer M, Salonen O, Pihko H, Norio R PEHO syndrome (progressive encephalopathy with edema, hypsarrhythmia, and optic atrophy): neuroradiologic findings. AJNR Am J Neuroradiol 1993; 14: 861–7. 8352158 PMC8333820

[awx155-B28] Tammenkoski M, Koivula K, Cusanelli E, Zollo M, Steegborn C, Baykov AA, Human metastasis regulator protein H-prune is a short-chain exopolyphosphatase. Biochemistry 2008: 47: 9707–13. 18700747 10.1021/bi8010847

[awx155-B29] Tekgül H, Tütüncüoğlu S Progressive encephalopathy with edema, hypsarrhythmia, and optic atrophy (PEHO syndrome) in a Turkish child. Turk J Pediatr 2000; 42: 246–9. 11105628

[awx155-B30] Zollo M, Ahmed M, Ferrucci V, Salpietro V, Asadzadeh F, Carotenuto M, Prune is crucial for normal brain development and mutated in microcephaly with neurodevelopmental impairment. Brain 2017; 140: 940–52. 28334956 10.1093/brain/awx014 PMC5382943

