# Supplementary material for: The phenotypic and molecular spectrum of PEHO syndrome and PEHO-like disorders
Source: Brain. 2017 Jul 12;140(8):e49. doi: 10.1093/brain/awx155 (PMC5806505; doi:10.1093/brain/awx155)
Supplement: Supplementary Table 1 [file brain-2017-00738-file004_awx155.pdf]

Supplementary Table 1 Diagnostic criteria for PEHO syndrome by Somer (1993)

**Necessary criteria**
Infantile (usually neonatal) hypotonia
Infantile spasms with myoclonic seizures
Profound psychomotor delay and severe hypotonia; lack of motor milestones and speech
Early loss (or absence) of visual fixation with atrophy of optic discs by age 2 years; normal ERG, extinguished VEP
Progressive brain atrophy on MRI, particularly in the cerebellum and brainstem; milder supratentorial atrophy
**Supportive criteria**
Distinctive facial features including narrow forehead, epicanthic folds, short nose, open mouth, receding chin
Peripheral edema (face and limbs), especially in early childhood
Brisk tendon reflexes in early childhood
Abnormal brainstem auditory evoked potentials
Absent cortical responses of somatosensory evoked potentials
Slow nerve conduction velocities in late childhood
Dysmyelination on magnetic resonance imaging
**Features that argue against PEHO syndrome**
Microcephaly at birth
Abnormal gyral formation on neuroradiological studies
Predominating spasticity in infancy
Reappearance of visual contact after cessation of infantile spasms
Hepato/splenomegaly or storage disorder on histological studies

ERG = electroretinogram; VEP = visual evoked potentials; PEHO = progressive encephalopathy with oedema, hypsarrhythmia, and optic atrophy.
